# Factors related to the psychological impact of malocclusion in adolescents

**DOI:** 10.1038/s41598-020-70482-4

**Published:** 2020-08-10

**Authors:** José Enrique Iranzo-Cortés, José Maria Montiel-Company, Carlos Bellot-Arcis, Teresa Almerich-Torres, Claudia Acevedo-Atala, José Carmelo Ortolá-Siscar, José Manuel Almerich-Silla

**Affiliations:** 1 Stomatology Department, University of Valencia, Valencia, Spain; 2 Stomatology Department, University of La Frontera, Temuco, Chile

**Keywords:** Dentistry, Quality of life

## Abstract

To study the association between orthodontic treatment need and the psychosocial impact of dental aesthetics in a sample of adolescents, as well as other associated factors. A transversal study was conducted on 1,158 adolescents (12–16 years old) examined at the schools selected for the 2018 epidemiological study on oral health in the Comunidad Valenciana (Spain). The need for orthodontic treatment was determined by DAI and IOTN. The psychosocial impact was established by the Psychosocial Impact of Dental Aesthetics Questionnaire (PIDAQ). Other variables considered were sex, social class, DMFT index and Body-Mass Index, and having previously worn or presently wearing an orthodontic device. A lineal regression statistical technique was applied to study the significant associations with the scoring obtained in PIDAQ. The PIDAQ scores revealed a significant and positive lineal relationship with regard to need for orthodontic treatment: DAI (ẞ = 0.20) and IOTN-DHC (ẞ = 4.87), in women (ẞ = 2.66) and a negative one for having previously worn an orthodontic device (ẞ = − 5.74). The rest of the variables had no statistical significance (*p* > 0.05). The psychosocial impact of dental aesthetics in adolescents is associated with the presence of malocclusion and the female sex, while the condition of having previously worn an orthodontic device reduces the psychosocial impact.

## Introduction

Currently, the demand for orthodontic devices is largely based on patients’ own perception of dental aesthetics. However, the diagnosis entailing the need for orthodontic treatment has traditionally been based on a normative and objective evaluation that considers cephalometric measurements that treat the pathology from a professional perspective. However, this approach barely considers the patients’ perception of their own malocclusion and how this aspect may affect their routine life, not only at a functional level, but also in terms of how their social relationships are impacted^1^.

In 2004, Hadam^2^ concluded that around 40% of patients requesting orthodontic treatment had been made fun of due to the appearance of their teeth, whereas there was no association between the degree of need for orthodontic treatment and the need perceived by the patients themselves. According to Kiekens et al.^3^, the objective of the patients that undergo an orthodontic treatment is to improve their dentofacial aesthetics to enhance their self-esteem and to feel socially accepted.

Multiple studies have shown an association between malocclusion and a greater psychosocial impact as the condition worsened^4–8^, revealing also that a correction of the malocclusion improved psychosocial impact results; the impact decreased after orthodontic treatment. Another factor studied was the role of sex as a variable that could affect the psychosocial impact on a patient finding that the psychosocial impact was greater in women in the studies that evaluated that aspect^5,7,9,10^. These findings justify the development of this study, evaluating how malocclusion can affect the self-perception of teenagers, and how other factors can improve or worsen the psychosocial impact.

One of the most widely used instruments in the evaluation of the psychosocial impact of malocclusions is the PIDAQ (Psychosocial Impact of Dental Aesthetics Questionnaire). This questionnaire measures quality of life aspects linked to oral health which are specifically linked to dental aesthetics. Devised by Klages et al. in 2006^11^, the questionnaire is filled in by the patient himself/herself and is geared towards use by adolescents and young adults; it has been widely validated in different languages and cultural contexts^12–15^.

The objective of this study was to evaluate the psychosocial impact that malocclusion has on adolescents aged between 12 and 16, as well as other factors that may influence the said psychosocial impact.

## Material and methodology

### Study design

A transversal study, both descriptive and analytical, was set up in the context of the 2018 Oral Health Study in the Spanish region known as Comunidad Valenciana. The sample size was established according to the prevalence of malocclusion in the 2010 study; the rates were: 20.9% at the age of 12 and 12.7% at the age of 15^16^, with a 95% confidence level and a precision level of 4%, resulting in a sufficiently large sample size of 687 patients to carry out the study.

### Calibration expertise of the examining dentists

The examinations were performed by three examiners, with calibration expertise in the use of IOTN (Index of Orthodontic Treatment Need) and DAI (Dental Aesthetics Index) and based on a Gold Standard. First, model-based theoretical sessions on calibration took place and the arising queries or doubts were clarified. Subsequently, a calibration exercise on 10 patients aged between 12 and 16 was conducted. All examiners obtained a high degree of diagnostic concordance in the use of the IOTN (weighted kappa > 0.85 in each examiner in relation to the Gold Standard) as well as in the use of DAI (Intra-class Correlation Coefficient > 0.85 in relation to the Gold Standard).

### Setting

A conglomerate-based sampling was carried out where 48 schools were selected from a total of 709 in the designated territory. At each school, between 20 and 25 pupils were randomly selected. The examinations took place on the premises of the selected schools between April and October 2018, as part of the Oral Health Study conducted throughout the region known as Comunidad Valenciana.

### Data gathering

To evaluate the orthodontic treatment need, measurements of malocclusion features were carried out to determine the DAI and the IOTN-DHC^17,18^. A flat, no 5 examination mirror and a probe gauged in millimetres was used: PCP 11.5B (Hu-Friedy); measurements were recorded in millimetres, thereby quantifying any malocclusion features. A bluish-white-spectrum light was used to correctly illuminate the oral cavity. To determine the IOTN-AC, a sheet was handed over with the proposed images of the index developer and those desired by the pupil himself/herself; this stage was made possible by using a mirror. The task was to identify which were the images that were believed to most closely represent the patient’s occlusion^18^. This last measurement was also carried out by an examiner. The caries were examined according to ICDAS II criteria during the intraoral examination. All findings were recorded on an examination sheet which also stated the adolescent’s date of birth, whether he/she was currently wearing an orthodontic device or had previously worn one, sex, and parents’ occupations.

The social class was determined according to parents’ occupation, using the British classification: UK Registrar-General’s Social Class which groups the population into 5 categories: I. Professionals and higher managerial and technical occupations; II. Lower managerial and technical occupations, trade; III: intermediate supervisory and clerical occupations; IV(a): skilled manual workers; IV(b): partly skilled manual workers; and V: unskilled workers^19^. For the present study, Categories I and II were designated as High Social Class, Category III was considered to be Middle Class, whereas Categories IV(a), IV(b) and V were considered as Working Class.

The questionnaire known as PIDAQ, validated by Montiel-Company and collaborators in 2013^13^ in its translation into Spanish, was given to the adolescents to be individually filled out. This questionnaire consisted of 23 items, formulated within a framework of both positive and negative interrogative approaches. Each item was evaluated according to the 5-point Likert scale ranging from 0 or zero (the item does not produce psychosocial impact on the quality of life of the patient) to 4 (the item produces maximum psychosocial impact on the quality of life of the patient)^11^. In order to calculate the overall score of the PIDAQ, items 1–6 were inverted, as their approach were positive and the rest of the items had a negative interrogative approach.

To determine the Body Mass Index (BMI), the participants were weighed, and their heights were recorded after which the formula: (weight (kg)/height (m)^2^) was applied.

### Ethical approval

This study was approved by the Ethics Committee of the University of Valencia (Reference number: H1510648717945) and complied with the recommendations of the Declaration of Helsinki. All participants gave informed consent, signed by their parents or legal guardians so that the intraoral examinations and questionnaires could be conducted.

### Statistical analysis

The data compiled was set out on a Microsoft Excel 2016 sheet and the statistical analysis was carried out by applying the IBM SPSS v. 24 programme.

The first stage of the statistical analysis involved devising descriptive statistics that determined means and proportions, with 95% confidence intervals, of all the variables of the study. To make comparisons between the means, the Student’s t-test and ANOVA variance were applied with post-hoc Bonferroni evaluation, as well as lineal tendency test. Finally, multivariate lineal regression models were applied using the step forward method to evaluate the relationship between the PIDAQ scores as the dependent variable. The predictor variables were DAI, IOTN-DHC and IOTN-AC, age, sex, social class, having previously worn an orthodontic device, DMFT and BMI. The level of statistical significance was determined as *p* < 0.05.

## Results

A total of 1,158 pupils between the ages of 12 and 15 participated in the study of which 995 underwent intraoral examination. The remainder of the group was not examined as they were wearers of an orthodontic device at the moment of the study, even though they had answered the questionnaire (PIDAQ).

Table 1 sets out the data for each of the variables registered; 14.1% of the pupils were wearing an orthodontic device at the moment of the study and hence were excluded from the determination of need for orthodontic treatment, giving a sample size of 995. Furthermore, 10.1% of the pupils presented a need for orthodontic treatment in accordance with the IOTN-DHC indices; the percentages were 5.9% for the IOTN-AC and 25.7% according to the DAI index. The mean value of PIDAQ for the whole of the sample was 21.1 (IC 95% = 20.3–21.9).

**Table 1:** Descriptive statistics of the sample.

Variable	Mean/percentage	CI 95%	Range
Age (n = 1,158)	14.60	14.45–14.64	12.14–16.64
**Sex (n = 1,158)**			
Male	47.67%	44.80–50.55%	–
Female	52.33%	49.45–55.20%	–
**Social class (n = 1,158)**			
Working class	23.32%	20.97–25.84%	–
Middle class	38.68%	35.92–51.53%	–
High social class	38.00%	35.25–40.83%	–
**Orthodontic treatment previously worn (n = 1,158)**			
No	70.03%	67.33–71.60%	–
At the moment of the study	14.08%	12.19–16.20%	–
Past	15.89%	13.90–18.11%	–
DMFT (n = 1,158)	0.91	0.82–0.99	0–10
BMI (n = 1,158)	21.67	21.45–21.88	14.11–41.34
**IOTN-DHC (n = 995)**			
1–2	69.25%	66.31–72.04%	–
3	20.70%	18.30–23.33%	–
4–5	10.05%	8.33–12.07%	–
**IOTN-AC (n = 995)**			
1–4	87.14%	84.91–89.07%	–
5–7	6.93%	5.52–8.68%	–
8–10	5.93%	4.62–7.57%	–
DAI mean (n = 995)	26.51	25.91–27.10	14.17–85.28
**DAI (n = 995)**			
13–25	59.20%	56.11–62.21%	–
26–30	15.08%	12.99–14.43%	–
31–35	12.86%	10.93–15.09%	–
36	12.86%	10.93–15.09%	–
PIDAQ total score (n = 1,158)	21.05	20.26–21.85	0–78
PIDAQ DSC (n = 1,158)	13.80	13.48–14.11	0–24
PIDAQ SI (n = 1,158)	3.56	3.28–3.85	0–30
PIDAQ PI (n = 1,158)	5.17	4.90–5.43	0–23
PIDAQ AC (n = 1,158)	2.12	1.96–2.28	0–12

Means and proportions CI 95%

The final PIDAQ score, as well as the Social Impact (SI), Psychological Impact (PI) and Aesthetic Concern (AC) domains indicated a significant positive lineal relationship with regard to the need for orthodontic treatment, being determined by DAI; the PIDAQ score increased as the need for orthodontic treatment increased. Furthermore, the Dental Self-Confidence (DSC) domain showed a significant negative lineal tendency, reflected in reduced confidence levels as the degree of malocclusion worsened (Table 2). The same significant associations were found when IOTN was the index used to determine the malocclusion; this result was the same regardless of the use of the dental health component (DHC) or the use of the aesthetic component, IOTN-AC, determined by the examiner (Table 3). The reliability of PIDAQ obtained a Cronbach’s alpha value of 0.91, whereas 0.90 was the value for the DSC domain, 0.84 for the SI domain, 0.83 for the PI and 0.80 for the AC.

**Table 2:** PIDAQ means for each grade of treatment need according to DAI (CI 95%) and ANOVA contrasts of means.

	Dental Aesthetic Index (DAI)				
	Values < 25 n = 589 Media (IC95%)	Values 26–30 n = 181 Media (IC95%)	Values 31–35 n = 97 Media (IC95%)	Values ≥ 36 n = 28 Media (IC95%)	ANOVA *p* value Linear trend *p* value
Dental Self-Confidence (DSC) Items 1–6	15.18 (14.78–15.58)	12.40 (11.63–13.17)	11.15 (9.95–12.36)	10.70 (9.73–11.68)	*p* < 0.01* *p* < 0.01
Social Impact (SI) Items 7–14	2.82 (2.48–3.17)	4.27 (3.53–5.02)	5.27 (3.97–6.57)	4.10 (3.12–5.08)	*p* < 0.01* *p* < 0.01
Psychological impact (PI) Items 15–20	4.31 (3.98–4.64)	6.03 (5.33–6.73)	7.01 (6.01–8.01)	6.54 (5.64–7.46)	*p* < 0.01* *p* < 0.01
Aesthetic Concern (AC) Items 21–23	1.87 (1.65–2.10)	2.56 (2.14–2.98)	2.34 (1.77–2.91)	2.34 (1.83–2.84)	*p* = 0.016* *p* = 0.105
PIDAQ Items 1–23	17.83 (16.86–18.79)	24.46 (22.53–26.41)	27.46 (24.32–30.60)	26.28 (23.57–29.00)	*p* < 0.01* *p* < 0.01

*p value < 0.05. Post Hoc Bonferroni. Statistical significance between groups: 1 versus 2, 3 and 4.

**Table 3:** PIDAQ means for each grade of treatment need according to IOTN-DHC (CI 95%), IOTN-AC (CI 95%) and ANOVA contrasts of means.

	IOTN-DCH				IOTN-AC			
	Grades 1–2 n = 689 Mean (CI 95%)	Grade 3 n = 206 Mean (CI 95%)	Grades 4–5 n = 100 Mean (CI 95%)	ANOVA *p* value Linear trend *p* value	Grades 1–4 n = 867 Mean (CI 95%)	Grades 5–7 n = 69 Mean (CI 95%)	Grades 8–10 n = 59 Mean (CI 95%)	ANOVA *p* value Linear trend *p* value
Dental Self-Confidence (DSC) Items 1–6	14.78 (14.39–15.16)	12.28 (11.54–13.01)	9.25 (8.25–10.25)	*p* < 0.01*^a^*p* < 0.01	14.33 (13.98–14.68)	10.00 (8.67–11.33)	8.86 (7.49–10.23)	*p* < 0.01*^d^ *p* < 0.01
Social Impact (SI) Items 7–14	2.99 (2.65–3.32)	4.36 (3.60–5.13)	5.17 (4.02–6.32)	*p* < 0.01*^b^ *p* < 0.01	3.20 (2.89–3.51)	5.12 (3.60–6.63)	5.80 (4.12–7.47)	*p* < 0.01*^d^ *p* < 0.01
Psychological impact (PI) Items 15–20	4.59 (4.28–4.91)	6.06 (5.38–6.74)	7.35 (6.33–8.37)	*p* < 0.01*^b^ *p* < 0.01	4.85 (4.55–5.14)	7.33 (6.21–8.45)	7.49 (5.98–9.01)	*p* < 0.01*^d^ *p* < 0.01
Aesthetic Concern (AC) Items 21–23	1.99 (1.78–2.20)	2.15 (1.76–2.53)	2.81 (2.22–3.40)	*p* = 0.02*^c^ *p* < 0.01	2.01 (1.82–2.20)	2.61 (1.89–3.32)	2.86 (2.11–3.62)	*p* = 0.024 *p* = 0.02
PIDAQ Items 1–23	18.79 (17.87–19.70)	24.29*^a^ (22.22–26.37)	30.08*^a^ (27.18–32.91)	*p* < 0.01*^a^ *p* < 0.01	19.75 (18.88–20.58)	29.06 (25.58–32.53)	31.29 (26.77–35.80)	*p* < 0.01*^d^ *p* < 0.01

*p value < 0.05.

Post Hoc Bonferroni. Statistical significance between groups: ^a^1 versus 2, 3 and 4; 2 versus 3 and 4; 3 versus 4, ^b^1 versus 2 and 3, ^c^1 versus 3, ^d^1 versus 2 and 3.

Table 4 sets out the differences between the PIDAQ scorings and its domains according to the variables studied. Significant differences were found with regard to sex, age, having previously worn an orthodontic device, and need for orthodontic treatment, in all the indices used for its determination: DAI, IOTN-DHC and IOTN-AC.

**Table 4:** Means of each PIDAQ domain and the total PIDAQ score according to sex, age, orthodontic treatment worn, in the past or at the moment of the study and social class (CI 95%).

Variable	PIDAQ DSC	PIDAQ SI	PIDAQ PI	PIDAQ AC	PIDAQ Total score
**Sex**					
Male n = 552	13.84 (13.42–14.25)	3.36 (2.97–3.76)	4.46 (4.13–4.80)	1.89 (1.68–2.11)	19.89 (18.90–20.87)
Female n = 606	13.76 (13.29–14.23)	3.75 (3.33–4.16)	5.81 (5.41–6.21)	2.33 (2.08–2.57)	22.12 (20.90–23.33)
*P* value	0.816	0.191	< 0.01**	0.01*	< 0.01**
**Age**					
12 n = 627	13.26 (12.83–13.69)	3.68 (3.29–4.06)	4.95 (4.59–5.31)	2.13 (1.91–2.36)	21.50 (20.45–22.55)
15 n = 531	14.43 (13.96–14.89)	3.43 (3.00–3.86)	5.43 (5.03–5.82)	2.10 (1.87–2.34)	20.53 (19.33–21.73)
*p* value	< 0.01**	0.396	0.08	0.857	0.232
**Orthodontic treatment worn at the moment of the study**					
No n = 995	13.71 (13.36–14.05)	3.49 (3.19–3.80)	5.17 (4.88–5.50)	2.10 (1.93–2.28)	21.06 (20.21–21.91)
Yes n = 163	14.35 (13.51–15.19)	4.01 (3.19–4.83)	5.13 (4.40–5.86)	2.23 (1.76–2.69)	21.02 (18.80–23.24)
*p* value	0.164	0.213	0.908	0.606	0.971
**Orthodontic treatment previously worn**					
No n = 974	13.29 (12.94–13.63)	3.71 (3.39–4.03)	5.48 (5.18–5.78)	2.22 (2.03–2.40)	22.13 (21.25–23.00)
Yes n = 184	16.49 (15.82–17.16)	2.77 (2.16–3.38)	3.50 (3.00–4.00)	1.61 (1.26–1.96)	15.39 (13.82–16.95)
*p* value	< 0.01**	< 0.01**	< 0.01**	< 0.01**	< 0.01**
**Social class**					
High n = 440	13.97 (13.47–14.47)	3.58 (3.10–4.05)	5.14 (4.70–5.57)	2.07 (1.81–2.34)	20.82 (19.55–22.08)
Middle n = 448	13.89 (13.37–14.41)	3.23 (2.80–3.65)	5.14 (4.72–5.57)	2.01 (1.75–2.27)	20.49 (19.21–21.77)
Working n = 270	13.36 (12.71–14.01)	4.10 (3.47–4.74)	5.25 (4.70–5.81)	2.38 (2.03–2.74)	22.38 (20.70–24.06)
*p* value	0.318	0.071	0.943	0.216	0.183
**Treatment need according to IOTN-DHC**					
No	14.20 (13.86–14.55)	3.30 (2.99–3.62)	4.93 (4.64–5.22)	2.02 (1.83–2.20)	20.05 (19.19–20.92)
Yes	9.25 (8.25–10.25)	5.17 (4.02–6.32)	7.35 (6.33–8.37)	2.81 (2.22–3.40)	30.08 (27.18–32.98)
*p* value	< 0.01**	< 0.01**	< 0.01**	< 0.01**	< 0.01**
**Treatment need according to IOTN-AC**					
No	14.01 (13.67–14.35)	3.35 (3.04–3.65)	5.03 (4.74–5.31)	2.05 (1.87–2.24)	20.42 (19.58–21.26)
Yes	8.86 (7.50–10.24)	5.80 (4.12–7.47)	7.49 (5.98–9.01)	2.86 (2.11–3.62)	31.28 (26.77–35.80)
*p* value	< 0.01**	< 0.01**	< 0.01**	0.03*	< 0.01**
**Treatment need according to DAI**					
No	14.59 (14.22–14.96)	3.12 (2.80–3.44)	4.69 (4.38–5.00)	2.00 (1.80–2.20)	19.22 (18.32–20.12)
Yes	12.39 (11.84–12.95)	4.35 (3.80–4.90)	6.01 (5.54–6.49)	2.33 (2.05–2.61)	24.84 (22.84–25.76)
*p* value	< 0.01**	< 0.01**	0.063	< 0.01**	< 0.01**

*significant *p* value < 0.05 **Highly significant *p* value < 0.01.

According to the lineal regression statistical analysis, DAI was a predictive value for the total value of PIDAQ, as it was for the DSC, SI and PI domains. The condition of having previously worn an orthodontic device was shown to be a predictive value for the DSC, PI, and AC domains. IOTN-DCH was a predictive value for the total of the questionnaire and for the DSC, PI, and AC domains. The parameter of female sex was a predictive value for the total of PIDAQ and for the PI and AC domains. Finally, IOTN-AC was a predictive value for the whole of the questionnaire and for the DSC and SI domains. The rest of the variables studied, (age, presently wearing an orthodontic device and social class) did not bring up statistical significance in the lineal regression models (Table 5).

**Table 5:** Linear regression models estimated for the PIDAQ scores and its domains: DSC, SI, PI and AC and their predictive variables.

	Model *p* value R^2^	Predictive variables	Beta coefficient (CI 95%)	*p* value
Total PIDAQ score	< 0.01** 0.114	DAI	0.20 (0.10–0.30)	< 0.01**
		Past orthodontic treatment worn	− 5.74 (− 7.84 to − 3.65)	< 0.01**
		IOTN-DHC	4.87 (1.63–8.11)	< 0.01**
		Female	2.66 (1.06–4.27)	0.02*
		IOTN-AC	4.43 (0.57–8.29)	< 0.01**
		Constant	14.68 (11.91–17.46)	
DSC	< 0.01** 0.158	DAI	− 0.11 (− 0.15 to − 0.07)	< 0.01**
		Past orthodontic treatment worn	2.72 (1.90–3.53)	< 0.01**
		IOTN-DHC	− 2.29 (− 3.56 to − 1.02)	< 0.01**
		IOTN-AC	− 1.95 (− 3.46 to − 0.44)	< 0.01**
		Constant	16.40 (15.36–17.44)	
SI	< 0.01** 0.02	IOTN-AC	1.82 (0.45–3.12)	< 0.01**
		DAI	0.04 (0.01–0.08)	0.01*
		Constant	2.21 (1.29–3.13)	
PI	< 0.01** 0.10	DAI	0.07 (0.04–0.10)	< 0.01**
		Female	1.52 (0.97–2.06)	< 0.01**
		Past orthodontic treatment worn	− 2.10 (− 2.83 to 1.37)	< 0.01**
		Age	0.31 (0.14–0.49)	< 0.01**
		IOTN-DHC	1.35 (0.32–2.39)	0.01*
		Constant	− 1.77 (− 4.49 to − 0.96)	
AC	< 0.01** 0.136	IOTN-DHC	0.75 (0.16–1.33)	< 0.01**
		Past orthodontic treatment worn	− 0.58 (− 1.03 to − 0.13)	0.01*
		Female	0.42 (0.07–0.77)	0.02*
		Constant	1.92 (1.65–2.19)	

## Discussion

Our results found a significant association between the PIDAQ scores and the need for orthodontic treatment determined by DAI and by considering both components of IOTN (IOTN-DHC and IOTN-AC), coinciding with findings in the studies by Al-Sarheed and Paula, respectively^20,21^. For each of the PIDAQ domains, the said association is maintained except for the Aesthetic Concern domain (AC). This result is also in line with those obtained by Almerich et al.^5^ and by Sardenberg et al.^12^. The adolescent population is more worried about appearance and aesthetics, aspects which are often not faced with objectivity and maturity, thereby having a possible influence on the results^12,22^. Further still, at times patients’ self-perception of their malocclusion is not related to the need for orthodontic treatment which is objectively established by the indices^22–24^. Manzanera et al. identified a low concordance between IOTN-AC and DAI (ICC = 0.16)^24^. The perception of the specialist is often very different and may lead to an understanding of the lack of association between the AC domain and the indices relative to need for orthodontic treatment^23^. A significant lineal relationship has been found between the scoring of the PIDAQ total and the need for orthodontic treatment determined by DAI and both components of the IOTN; this situation tends to increase the PIDAQ scorings as the need for orthodontic treatment increases. These results coincide with those obtained in other studies^21,25,26^, thereby confirming that PIDAQ indicates a correlation with the aesthetic impact of malocclusion. Klages et al.^11,27^, found that malocclusion may have a psychological impact on adolescents, to such an extent that their self-confidence and social interactions are affected. Adolescents are particularly concerned about the appearance of their teeth; it follows then that an adequate oral health and the correct alignment of teeth are of paramount importance in one’s own facial perception. This corroborate the theory that dentofacial aesthetics plays a major role an important role in social interaction and psychological wellbeing^22,28,29^.

Due to the impact that malocclusions have on the quality of life related to oral health, research in this field is focusing on patients’ own perception of their own body-image during the planning stages of orthodontic treatments^1,30^. The use of instruments to measure the psychosocial impact of malocclusion needs to be assessed within the framework of normative indices when determining orthodontic treatment need^31,32^. However, the psychosocial impact is variable; on the one hand, there are patients with severe malocclusions who feel either satisfied or indifferent with their dental aesthetics. On the other, there are those who are very concerned about light irregularities which are inconsequential^32–34^.

According to O’Brien et al.^35^, the most significant impact of malocclusion in patients’ quality of life is reflected in the psychosocial dimension instead of feeling a lack of satisfaction with the function. For patients wearing orthodontic devices, the emotional and social domains (which include aspects such as embarrassment, feeling ill at ease and avoidance of smiling) are the most relevant.

Following on from this, malocclusion does not always negatively affect patients’ quality of life; its impact will depend on expectations, preferences, economic resources or the psychological profile of the individual, along with social and cultural values of the socio-economic setting they live in^36–38^.

In some cases, patients’ dissatisfaction in dental aesthetics may have an impact on their satisfaction with their appearance, leading to their social relationships being affected^27,34,39,40^. Under this rationale, among the benefits of orthodontic treatment that would normally be expected, mention must be made of the improvement in self-esteem and the reduction of anxiety in conducting social relationships^27,41^.

Some authors^25,42,43^, consider that it is more appropriate to analyse the psychosocial impact of dental aesthetics in adults as they have a more advanced and stable emotional development; they also have a more realistic perception of aesthetics than adolescents. Furthermore, other authors such as Tuominen et al. in 1995 and Cooper et al. in 2000, observed that the perception of aesthetics is subject to variation and may even improve with age, thereby concluding that need for orthodontic treatment appears to diminish with time, even if the patient does not undergo orthodontic treatment^44,45^.

The results of our study show that there are statistically significant differences in the psychosocial impact of malocclusion as a function of sex, being greater in the case of women than in men. The results fall in line with those obtained by authors such as Birkeland et al. in 1996, Al-Shareed et al. in 2003 and Hamdan et al. in 2004^2,20,30^, indicating that women are more critical in their perception of the impact related to dental aesthetics dental, given that in the need for objective treatment based on occlusal differences, there were no significant sex differences. Some authors suggest that this may be explained by the overall greater concern by women about health issues compared to men. This is reflected by greater attention being paid to health, a greater awareness of the impact of oral health, the role of facial beauty and considerations pertaining to the quality of life^33–35,46^.

The regression analysis revealed that the need for orthodontic treatment, determined by DAI or IOTN were in fact predicting variables for a greater psychosocial impact and of the majority of its components, while the condition of previously having worn an orthodontic device predicted a lower psychosocial impact. No association was found between the PIDAQ scoring and other variables such as age, indices of caries, social-class, BMI, or the condition of currently wearing an orthodontic device. The variables female sex and the age of the pupil showed an association with some of the PIDAQ components or with the total scoring. The results herein are similar to those obtained in other studies^5^.

As concluding remarks, malocclusion is significantly associated with a greater psychological impact in adolescents due to their perception of dental aesthetics. Having previously worn an orthodontic device in the past has been reflected in the lower scores given in the questionnaire, while psychosocial impact scores were significantly higher in the female sex.
